# Clues to Disease Activity in Juvenile Dermatomyositis: Neopterin and Other Biomarkers

**DOI:** 10.3390/diagnostics12010008

**Published:** 2021-12-21

**Authors:** Amer Khojah, Gabrielle Morgan, Lauren M. Pachman

**Affiliations:** 1 Division of Pediatric Rheumatology, Ann & Robert H. Lurie Children’s Hospital of Chicago, Chicago, IL 60611, USA; pachman@northwestern.edu; 2 Division of Allergy & Immunology, Ann & Robert H. Lurie Children’s Hospital of Chicago, Chicago, IL 60611, USA; 3 Feinberg School of Medicine, Northwestern University, Chicago, IL 60611, USA; 4 Cure JM Center of Excellence, Stanley Manne Research Center, Chicago, IL 60611, USA; GAMorgan@luriechildrens.org

**Keywords:** neopterin, Juvenile Dermatomyositis, disease activity scores, TNFα-308A polymorphism, CXCL11, CXCL10, NK cell

## Abstract

Easily accessible biomarkers are urgently needed to evaluate immune activation in Juvenile Dermatomyositis (JDM). The goal of this retrospective study is to define immunological and clinical differences between untreated JDM patients with either normal or elevated (>10 mmol/L) levels of neopterin, a biomarker of macrophage activation. We included all JDM with neopterin data obtained before initiating medical therapy. We assessed T, B, NK cell populations, muscle enzymes, and disease activity scores for skin (sDAS), muscle (mDAS), total (tDAS), the duration of untreated disease, disease course, and myositis-specific antibody (MSA). Seventy-nine percent of 139 untreated JDM patients had elevated serum neopterin. The group with elevated neopterin had significantly more active disease: tDAS 11.9 vs. 8.1 (*p* < 0.0001), mDAS 5.8 vs. 3.1 (*p* < 0.0001), sDAS 6.1 vs. 4.9 (*p* = 0.0002), aldolase 24.0 vs. 7.6 U/L (*p* < 0.0001), von Willebrand factor antigen (*p* < 0.0001), and ESR 19.8 vs. 11.5 mm/hr (*p* = 0.01). The flow cytometry documented both reduced T cells (1494 vs. 2278/mm^3^, *p* = 0.008) and NK cells (145 vs. 240/mm^3^, *p* = 0.003). TNFα-308AA/AG polymorphism was more common in children with elevated neopterin than TNFα-308GG (*p* 0.05). We conclude that the availability of neopterin data will contribute to the rapid assessment of untreated JDM disease activity.

## 1. Introduction

Our recent RNA-Seq studies documented that although children with JDM appear to have inactive disease, prompting reduction of medication, they have upregulated transcriptional activity [1]. This study was designed to determine if an easily available biomarker, such as neopterin, might be a serological clue to disease activity. Neopterin is a metabolic product of guanosine triphosphate, which is produced by macrophages upon stimulation with interferon-gamma (IFN-γ) from activated T helper-1 cells (Th1) [2]. Therefore, the degree of macrophage activation can be assessed by measuring neopterin in body fluids such as serum, urine, synovial fluid, or cerebrospinal fluid [3,4]. Increased serum neopterin level is seen in hemophagocytic lymphohistiocytosis syndrome, chronic infection, and autoimmune disease [2,5,6,7]. Juvenile Dermatomyositis (JDM) is a rare pediatric systemic autoimmune disease characterized by skin rash and muscle damage [8]. JDM’s annual incidence in the United States is 2.7–3.4 per million with a mean age of diagnosis of 7.3 years for boys and 6.7 years for girls [9]. Previously, the diagnosis of JDM was made based on the Bohan and Peter criteria which includes classic skin rash, symmetrical muscle weakness, elevated muscle enzyme, muscle biopsy, and electromyographic finding [10,11,12]. However, in 2017, EULAR and ACR proposed new diagnostic criteria which refined the scoring for each criterion and added esophageal dysmotility and anti-histidyl-tRNA synthetase antibody, leading to improvement in the specificity over the original Bohan and Peter criteria [12]. Others had suggested including MRI findings, nailfold capillary changes, and other myositis-specific antibodies (MSA) to the criteria to further improve the diagnostic yield and minimize the need for muscle biopsy in the often-young child [13].

The pathophysiology of JDM is complex and not completely understood. In addition to perifascicular muscle fiber atrophy, there is increased lymphocytes (CD3+ve and CD19+ve) and plasmacytoid dendritic cell infiltration in the untreated JDM child’s muscle biopsy [8,12]. The plasmacytoid dendritic cell is a major source of type 1 interferon (IFN-α and β) production. Type 1 interferons play an important role in natural killer (NK) cell function by promoting cell proliferation, cytotoxicity, and IFN-γ production [14,15]. A preliminary study in pediatric orbital myositis showed a possible link between decreased peripheral blood NK cell counts and increased disease activity [16]. Furthermore, IFN-α promotes CD4 Th1 differentiation [17]. In contrast, IFN-γ stimulates macrophage activation and production of neopterin, TNF α, and IL-12, leading to further propagation of Th1 differentiation and immune activation.

Despite the initial studies showing elevated neopterin in children with active Juvenile Dermatomyositis [18,19], the adoption of neopterin as a biomarker in clinical practice still appears to be limited to confirming macrophage activation in diseases such as hemophagocytic lymphohistiocytosis syndrome [6]. The goal of this study was to determine the utility of neopterin as a potential serologic indicator of disease activity and study the clinical and immunological differences between newly diagnosed, untreated JDM patients with elevated sera levels of neopterin compared to those with normal levels of neopterin.

## 2. Materials and Methods

### 2.1. Subjects

This retrospective chart review study was approved by the Institutional Review Board (IRB) at Ann & Robert H. Lurie Children’s Hospital of Chicago (IRB 2008-13457) on 12 September 2008 and was reviewed annually with the last renewal on 11 October 2021. Inclusion criteria included all children who were seen at Lurie Children’s Hospital between 1980 and 2021, who met the Bohan and Peter criteria [7,8] for definite or probable JDM, who had serum neopterin level before initiating medical therapy, and who signed an informed consent for the study. In addition, we excluded subjects with overlap syndrome (such as positive anti-U1 RNP, anti-U2 RNP, or anti-PM-Scl) from the analysis.

A total of 139 children with JDM (78% female, 22% male) were included. The racial and ethnic background is as follows: White, Non-Hispanic—74%; White, Hispanic—16%; African American—4%; Asian—3%; and Others—3%. The mean age at enrolment was 6.8 years (+/−3.6 SD). The mean duration of untreated disease was 10.8 months (+/−17.5 SD). Disease group by myositis-specific antibodies (MSA) [13]: 29.5% anti-P155/140 (Anti-TIF1- γ), 7% anti-Mi2, 6% both anti-Mi2 and anti-P155/140 (Anti-TIF1- γ), 5% anti-MJ (Anti-NXP-2), 1.5% anti-MDA5 (anti-CADM140), and 26% MSA negative. Of note, 24% of the 139 cases were seen before the presence of MSA was identified. The demographic data are presented in Table 1. For the 69 subjects who have sequential data over 36 months, we assessed neopterin level and the disease activity score at three time points: baseline/untreated, 2–3 months after treatment, and first visit off steroid therapy (typically 1–2 years later).

### 2.2. Disease Activity Assessment

The disease activity score total (tDAS) is based on observations related to the child’s skin rash (sDAS) and muscle weakness (mDAS) and is employed to assess disease activity in all JDM patients [20]. The Childhood Myositis Assessment Scale (CMAS) [21] was independently assessed by a certified physical therapist. The following muscle enzymes were measured before treatment and at every visit: creatine phosphokinase (CK), lactate dehydrogenase (LDH), aspartate aminotransferase (AST), and aldolase. We also measured the erythrocyte sedimentation rate (ESR) and von Willebrand factor antigen as potential indicators of disease activity. The number of nailfold capillary end row loops (ERL) was assessed by averaging the number of end row capillary loops per mm in the eight digits excluding thumbs [22]. The disease course was designated as *monophasic* if the child completed therapy without a subsequent disease flare, and *polyphasic* when a patient had completed therapy but had a subsequent recurrence of disease requiring re-initiation of medication at any time during their disease course. Children with a *chronic* disease course had at least 36 months of data available documenting active symptoms and had not yet completed therapy. The disease course was designated as “*unknown*” when patients lacked enough follow-up data to determine the type of disease course.

### 2.3. Methods

The neopterin level was measured by a competitive enzyme-linked immunosorbent assay (ALPCO diagnostics kit) in the clinical immunology lab at the Ann & Robert H. Lurie Children’s Hospital of Chicago. Elevated serum neopterin was defined by levels ≥ 10 nmol/L. The following cells surface markers were assessed by flow cytometry: CD3, CD4, CD8, CD19, and CD16/CD56, to determine T, B, and NK cell populations, respectively. Myositis-specific antibodies [23] were measured via immunoprecipitation and immunodiffusion at Oklahoma Medical Research Foundation. The TNF-α alleles at −308 primers and probe used to detect alleles (AA, AG, and GG) were synthesized at the Northwestern University Biotechnical Facility, Chicago, Illinois [24]. Meso Scale Discovery^®^ technique [25] was used to measure the serum level of C-X-C motif chemokine 11 (CXCL11)/interferon-inducible T-cell alpha chemoattractant (I-TAC), C-X-C motif chemokine 10 (CXCL10)/interferon gamma-induced protein 10 (IP-10), and angiopoietin-2 from 11 untreated JDM patients.

### 2.4. Statistical Analysis

IBM SPSS Statistics 26^®^ software was used to perform Student’s *t*-test and chi-square to compare the baseline characteristics and disease activity markers of children with untreated JDM who had normal levels of neopterin compared with those who had elevated neopterin. We also used Pearson correlation to explore the relationship between various disease activity scores and serum neopterin levels in untreated JDM subjects. Graphpad Prism 8 software generated the figures.

## 3. Results

**Figure 1:**
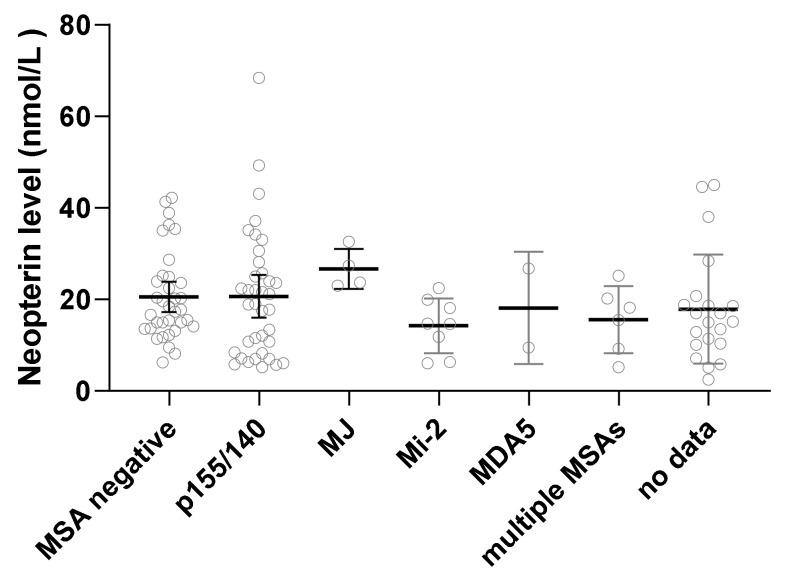
Myositis-specific antibodies and neopterin level. The serum neopterin level varied slightly based on the various MSA (myositis-specific antibodies) groups; however, the difference was not statistically significant by one way ANOVA test. The anti-MJ (Anti-NXP-2) group had the highest mean neopterin at 24.8 +/− 8.1 SD nmol/L, and the Mi2 group had the lowest level at 14.7 +/− 5.50 SD nmol/L.

The mean serum neopterin level for all untreated JDM (*n* = 139) was 19 nmol/L +/− 11.4 SD; the normal neopterin range is 10 nmol/L and below. The median neopterin level was 17.1 nmol/L with levels ranging from 2.4 nmol/L up to 68.4 nmol/L. With respect to the impact of the different MSAs groups, the anti-MJ (Anti-NXP-2) group had the highest mean neopterin at 24.8 +/− 8.1 SD nmol/L, and the Mi2 group had the lowest level at 14.7 +/− 5.50 SD nmol/L; however, the difference was not statistically significant (Figure 1).

The study subjects were divided into two groups based on their serum neopterin level: 79% with elevated neopterin and 21% with normal neopterin. The elevated neopterin group had a significantly shorter duration of untreated disease (*p* = 0.03). The group with elevated neopterin had significantly more active disease with mean tDAS 11.9 vs. 8.1 (*p* < 0.0001), sDAS 6.1 vs. 4.9 (*p* = 0.0002), mDAS 5.8 vs. 3.1 (*p* < 0.0001), and CMAS 30 vs. 40.4 (*p* = 0.007). Of note, the record of a lower CMAS is associated with more muscle weakness and therefore more disease activity. Muscle enzymes (aldolase, LDH, and AST) and von Willebrand factor antigen were significantly higher in the group with elevated neopterin (Table 2). The JDM children with elevated neopterin had a higher ESR 19.8 vs. 11.5 mm/hr (*p* = 0.01), but the values for both groups were within the “normal” range (<20 mm/hr). The type of disease course appeared not to be associated with the initial neopterin level. Of note, only 30% of the untreated JDM children had elevated ESR (>20 mm/hr), the conventional indicator of inflammation.

**Figure 2:**
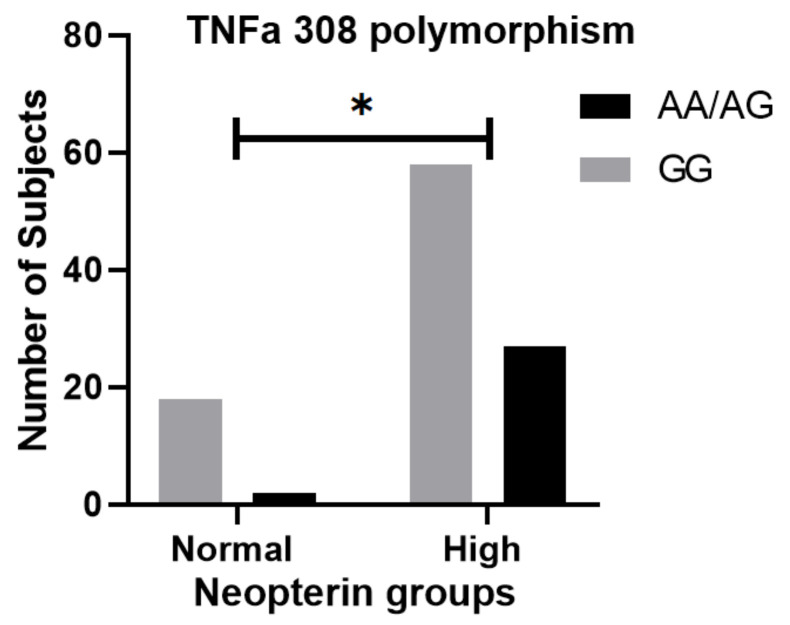
TNFα-308AA/AG polymorphism and neopterin level. TNFα-308AA/AG polymorphism was more common in children with elevated neopterin than TNFα-308GG (chi-square, *p* = 0.05). Of note, * means *p* < 0.05.

**Figure 3:**
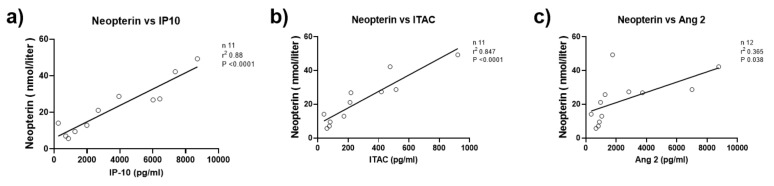
Serum neopterin levels are highly correlated with other biomarkers of disease activity: IP-10 (CXCL10), I-TAC, and angiopoietin 2 (Ang2). (**a**) Shows a strong correlation between serum neopterin level and with CXCL10 (IP-10: interferon gamma-induced protein 10) in untreated JDM subjects (r^2^ = 0.88, *p* < 0.0001). (**b**) Shows a strong correlation between serum neopterin level and CXCL11 (I-TAC: interferon-inducible T-cell alpha chemoattractant) in untreated JDM subjects (r^2^ = 0.85, *p* < 0.0001). (**c**) Shows significant correlation between angiopoietin 2 and serum neopterin level (r^2^ = 0.37, *p* = 0.038).

The flow cytometry of children with untreated JDM with elevated serum neopterin showed a statistically significant reduction in the absolute total T cell count (1494 vs. 2278/mm^3^, *p* = 0.008), CD4+ T cells (1004 vs. 1533/mm^3^, *p* = 0.009), CD8+ T cell (463 vs. 672/mm^3^, *p* = 0.019), and NK cells (145 vs. 240/mm^3^, *p* = 0.003), but not B cells (748 vs. 924/mm^3^, *p* = 0.097) (Table 2). TNFα-308AA/AG polymorphism was more common in children with elevated neopterin than TNFα-308GG (chi-square, *p* = 0.05) (Figure 2). For a small subset of untreated JDM patients (*n* = 11), serum neopterin level correlated strongly with CXCL10 (IP-10) (R^2^ = 0.88 *p* < 0.0001), as well as other biomarkers, CXCL11 (I-TAC) (R^2^ = 0.85 *p* < 0.0001), and to a lesser degree, angiopoietin 2 (R^2^ = 0.37 *p* = 0.038) (Figure 3).

**Figure 4:**
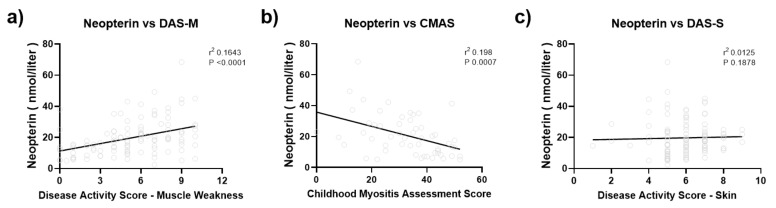
Correlation between serum neopterin level and clinical disease activity markers. (**a**) There was a correlation between the serum neopterin level and muscle weakness disease activity score (mDAS) (r^2^ = 0.16, *P* < 0.0001) and (**b**) Childhood Myositis Assessment Scalee (CMAS) (r^2^ = 0.2, *P* = 0.0007), (**c**) but not skin disease activity score (sDAS) (r^2^ = 0.01, *P* = 0.19).

**Figure 5:**
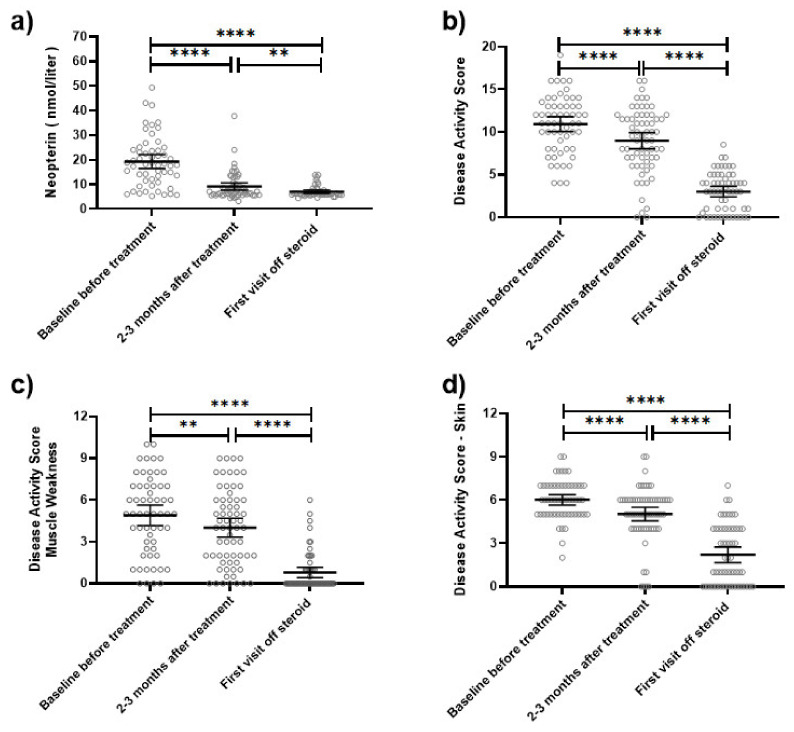
Serum neopterin level and disease activity scores (DAS) through disease course. For the 69 subjects with a sequential date over 36 months, we conducted paired *t*-tests to compare mean neopterin levels and disease activity scores across 3 time points: baseline/untreated, 2–3 months after treatment, and first visit off steroid therapy. (**a**) The mean serum neopterin level decreased from 19.3 nmol/L to 9.12 nmol/L (*p* < 0.0001 by paired *t*-test) 2–3 months after treatment. This was followed by a further reduction to 7.0 nmol/L in the first visit off steroid therapy (*p* = 0.01 by paired *t*-test compared to the 2nd time point). (**b**) Total disease activity scores (tDAS) decreased over the three-assessment time point: the mean tDAS scores were 10.9, 9.0, and 3.0, respectively. (**c**) Muscle weakness disease activity scores (mDAS) decreased over the three-assessment time point with means of 4.9, 4.0, and 0.8, respectively. (**d**) Skin disease activity scores (sDAS) decreased over the three-assessment time point with means of 6.0, 5.0, and 2.2, respectively. Of note, ** means *p* < 0.01 and **** means *p* < 0.0001.

There was a positive correlation between the serum neopterin level and disease activity markers, tDAS (R^2^ = 0.14 *p* < 0.0001), mDAS (R^2^ = 0.16 *p* < 0.0001), and CMAS (R^2^ = 0.2 *p* = 0.0007), but not sDAS (R^2^ = 0.01 *p* = 0.19) (Figure 4). For the 69 subjects with longitudinal data, serum neopterin decreased dramatically after 2–3 months of medical therapy (mean reduction of 9 nmol/L and *p* < 0.0001 on paired *t*-test) as the muscle weakness slowly improved (Figure 5).

## 4. Discussion

The elevated serum neopterin in nearly 80% of untreated JDM argues for the importance of Th1 cells activation and IFN-γ production in the majority of JDM patients. Elevated serum neopterin patients have more active disease—higher DAS and lower CMAS—consistent with prior studies [19]. Although reports from Japan and China document anti-MDA5 (anti-CADM140) dermatomyositis to have the most elevated serum neopterin level, especially in patients with severe lung disease [26,27], there was not a statistically significant difference in neopterin level between the different MSAs in our cohort. This discrepancy is severely influenced by the fact that we have a small number of MDA5 positive patients in our study (only two patients have MDA5 antibody) and that interstitial lung disease is also associated with an increased neopterin [28]. In our cohort, MJ-positive (Anti-NXP-2) JDM patients had the highest serum neopterin (mean of 24.8 +/− 8.1 SD nmol/L) and the highest tDAS (mean 11.8 ± 4 SD) and lowest CMAS (mean 26 ± 7.1 SD). JDM patients with TNFα-308A allele have prolonged disease courses and increased risk of pathologic calcification [24]. Peripheral blood mononuclear cells and muscle fibers from TNFα-308AA-positive children with JDM make more TNFα in comparison to those who are positive for TNFα-308GG [24,29]. Hence, it is not surprising to have higher neopterin production in patients with TNFα-308AA, as shown in our cohort. The immunological phenotype of patients with elevated neopterin includes lower T and NK cells. We speculate that the reduction in the peripheral T and NK cells could be a consequence of the migration of these cells to the inflamed tissue because of the greater degree of muscle weakness and elevated muscle enzymes in children with elevated neopterin. Future histologic studies are needed to verify this hypothesis. Of note, lymphocytic infiltration and the formation of follicle-like structures have been documented in JDM muscle in cases with severe muscle involvement [30].

Other serum biomarkers in the untreated JDM child with active disease are: CXCL11 (I-TAC), CXCL10 (IP-10), and angiopoietin 2 [25]. Biomarkers obtained at diagnosis of JDM that appear to have predictive value for a prolonged disease course include elevated galectin-9, CXCL10, and TNFRII (*p* < 0.05) [31]. These biomarker-based clusters do not appear to be dependent on the child’s MSA serotypes [25]. The data for other biomarkers in JDM include documentation that the JDM child’s response to corticosteroids mimics that of adults with ANCA-positive vasculitis [32]. In addition to serum-accessible biomarkers, data from RNA-Seq [1] and nailfold capillaries [33] are usually abnormal when obtained from the child with occult disease activity in the presence of “clinical quiescence”. In the present study, RNA-Seq was not obtained. There was no association of neopterin with the number of end row capillary loops, which take a longer time (greater than 2 months after disease onset) to “drop out” [34].

The limitations of this study include the following: First, 24% of the JDM samples, those obtained prior to 2007, when MSA data were first available, are unknown. Second, the study was not powered enough to examine the effect of possible confounders, such as MSA group, child gender, or ethnicity, which can affect initial serum neopterin level. Finally, the study did not include data on preceding viral infections such as COVID-19, which can increase serum neopterin level.

In conclusion, neopterin is a useful biomarker that assesses macrophage activation and correlates with disease activity in untreated JDM children. Furthermore, serum neopterin level improves relatively quickly after initiation of effective immunosuppression, making this potential biomarker appealing to assess the response of immunologic activity to therapy in non-specialty care centers. Finally, elevated neopterin is associated with TNFα-308A polymorphism as well as decreased absolute NK and T cell counts.

## Figures and Tables

**Table 1:** Neopterin levels in 139 untreated children with JDM: demographic characteristics.

	Elevated Serum Neopterin Group	Normal Serum Neopterin Group	*p*-Value
Number of subjects	110	29	
**Age at onset of symptoms in years (mean/SD)**	6.97	6.81	0.832
**Duration of untreated disease in months (mean/SD)**	**8.19**	**20.63**	**0.030**
**Gender**			
Female	84 (76%)	24 (83%)	0.462
Male	26 (24%)	5 (17%)	
**Race/ethnicity**			
White, Non-Hispanic	79 (72%)	23 (79%)	0.840
White, Hispanic	18 (16%)	4 (14%)	
African American	5 (5%)	1 (3%)	
Asian	4 (4%)	0 (0%)	
Others	3 (3%)	1 (3%)	
**Myositis-specific antibodies**			
P155/140	30 (27%)	11 (38%)	0.167
MJ	7 (6%)	0 (0%)	
Mi2	8 (7%)	2 (7%)	
MDA5	1 (1%)	1 (3%)	
Multiple MSAs	4 (4%)	4 (14%)	
Negative	33 (30%)	4 (14%)	
Not done	26 (24%)	7 (24%)	
**Disease course**			
Monophasic	48 (44%)	13 (45%)	0.674
Polyphasic	15 (14%)	6 (21%)	
Chronic	21 (19%)	3 (10%)	
Unknown	26 (24%)	7 (24%)	

**Table 2:** Neopterin levels in 139 untreated children with JDM: disease activity markers and flow cytometry results.

Clinical Findings	Elevated Serum Neopterin Group	Normal Serum Neopterin Group	*p*-Value
**Clinical disease activity indicator**			
Disease activity score—total	**11.92 ± 3.19**	**8.13 ± 3.57**	**<0.0001**
Disease activity score—skin	**6.06 ± 1.47**	**4.86 ± 1.58**	**0.0002**
Disease activity score—muscle weakness	**5.84 ± 2.69**	**3.13 ± 2.89**	**<0.0001**
Childhood Myositis Assessment Scale (CMAS)	**30.05 ± 12.60**	**40.43 ± 9.94**	**0.007**
Nailfold capillary end row loops (ERL)	4.86 ± 1.70	4.99 ± 1.25	0.749
**Laboratory disease activity indicator**			
Erythrocyte sedimentation rate (ESR)	**19.8 ± 14.63**	**11.55 ± 9.03**	**0.01**
von Willebrand factor antigen	**171.1 ± 79.68**	**109.15 ± 57.23**	**<0.0001**
**Muscle enzymes**			
Creatine phosphokinase (CK)	2486.51 ± 7494.67	724.81 ± 3116.15	0.244
Aspartate aminotransferase (AST)	**136.45 ± 224.89**	**53.96 ± 103.02**	**0.008**
Lactate dehydrogenase (LDH)	**520.23 ± 407.79**	**273.19 ± 153.15**	**<0.0001**
Aldolase	**24 ± 36.99**	**7.59 ± 3.47**	**<0.0001**
**Flow cytometry**			
Total T cells (CD3+)	**1494.36 ± 673.79**	**2278.43 ± 1264.74**	**0.008**
T helper cells (CD3+ CD4+)	**1004.06 ± 463.22**	**1533.22 ± 869.07**	**0.009**
T cytotoxic cells (CD3+ CD8+)	**462.97 ± 251.984**	**671.61 ± 379.72**	**0.019**
B cells (CD19+)	747.81 ± 424.01	924.39 ± 550.99	0.097
NK cells (CD16+/CD56+)	**144.94 ± 124.67**	**240.13 ± 159.41**	**0.003**

## Abbreviations

JDM: Juvenile Dermatomyositis, Th1: T helper-1 cells, NK: natural killer cell, INF: interferons, DAS: disease activity score, mDAS: muscle weakness disease activity score, sDAS: skin disease activity score, ERL: capillary end row loops, MSA: myositis-specific antibodies, CMAS: Childhood Myositis Assessment Scale, LDH: lactate dehydrogenase, AST: aspartate aminotransferase, CK: creatine phosphokinase, ESR: erythrocyte sedimentation rate, ERL: nailfold capillary end row loops. CXCL11: C-X-C motif chemokine 11, I-TAC: interferon-inducible T-cell alpha chemoattractant, CXCL10: C-X-C motif chemokine 10, IP-10: interferon gamma-induced protein 10, and Ang2: angiopoietin 2.
